# Excess Soluble CD40L Contributes to Blood Brain Barrier Permeability *In Vivo*: Implications for HIV-Associated Neurocognitive Disorders

**DOI:** 10.1371/journal.pone.0051793

**Published:** 2012-12-12

**Authors:** Donna C. Davidson, Michael P. Hirschman, Anita Sun, Meera V. Singh, Karl Kasischke, Sanjay B. Maggirwar

**Affiliations:** 1 Department of Microbiology and Immunology, University of Rochester School of Medicine and Dentistry, Rochester, New York, United States of America; 2 Department of Neurology, University of Rochester School of Medicine and Dentistry, Rochester, New York, United States of America; 3 Department of Neurology, University of Ulm Medical Center, Ulm, Germany; University of Nebraska Medical Center, United States of America

## Abstract

Despite the use of anti-retroviral therapies, a majority of HIV-infected individuals still develop HIV-Associated Neurocognitive Disorders (HAND), indicating that host inflammatory mediators, in addition to viral proteins, may be contributing to these disorders. Consistently, we have previously shown that levels of the inflammatory mediator soluble CD40L (sCD40L) are elevated in the circulation of HIV-infected, cognitively impaired individuals as compared to their infected, non-impaired counterparts. Recent studies from our group suggest a role for the CD40/CD40L dyad in blood brain barrier (BBB) permeability and interestingly, sCD40L is thought to regulate BBB permeability in other inflammatory disorders of the CNS. Using complementary multiphoton microscopy and quantitative analyses in wild-type and CD40L deficient mice, we now reveal that the HIV transactivator of transcription (Tat) can induce BBB permeability in a CD40L-dependent manner. This permeability of the BBB was found to be the result of aberrant platelet activation induced by Tat, since depletion of platelets prior to treatment reversed Tat-induced BBB permeability. Furthermore, Tat treatment led to an increase in granulocyte antigen 1 (Gr1) positive monocytes, indicating an expansion of the inflammatory subset of cells in these mice, which were found to adhere more readily to the brain microvasculature in Tat treated animals. Exploring the mechanisms by which the BBB becomes compromised during HIV infection has the potential to reveal novel therapeutic targets, thereby aiding in the development of adjunct therapies for the management of HAND, which are currently lacking.

## Introduction

CD40L (also known as CD154), a type II membrane glycoprotein of the tumor necrosis factor (TNF) family, is a co-stimulatory molecule found on T cells, B cells, and platelets, well known for its classical role in stimulating antigen presenting cells [1]. However, upon cleavage from the cell surface, a truncated, soluble form (sCD40L) is released, which retains its ability to form trimers and engage its receptor, CD40 [2]. It has been suggested that platelets produce approximately 95% of all sCD40L found in plasma [3], which is released upon their stimulation, thus implicating platelets as a major component in a variety of inflammatory disorders in which sCD40L is indicated.

Increased numbers of activated platelets have been reported in human immunodeficiency virus type-1 (henceforth referred to as HIV)-infected individuals [4], [5], while platelet decline has been proposed to predict brain injury and increased risk of developing HIV-Associated Neurocognitive Disorders (HAND) [6], [7], suggesting that increased consumption may be the consequence of aberrant platelet activation during infection. Consistently, we previously demonstrated an increase in sCD40L in both plasma and cerebrospinal fluid (CSF) of cognitively impaired, HIV-infected individuals as compared to HIV-infected, non-cognitively impaired counterparts [8]. Furthermore, our group recently demonstrated that treatment of brain microvascular endothelial cells (BMVECs) with sCD40L resulted in upregulation of adhesion molecules and led to an increase in monocyte adhesion to these cells in an *in vitro* model of the blood brain barrier (BBB) [9]. Therefore, we hypothesized that accumulation of sCD40L in HAND patients may be contributing to increased BBB permeability, thereby providing pro-inflammatory leukocytes an avenue of descent into the CNS.

Under physiologic conditions, the BBB serves to protect the brain from circulating pathogens or toxins in a highly ordered fashion [10]. However, deterioration or alterations of the BBB can lead to the development of many neurological complications, not only in the context of HIV infection [11], [12], but also in a wide range of other disorders, collectively termed vascular dementia or vascular cognitive impairment [13]. Consistently, increased infiltration of the CNS by activated leukocytes is widely believed to be one of the largest contributing factors in the progression of HAND, due to development of a pro-inflammatory, progressively neurotoxic environment [12]. Furthermore, despite the relatively widespread use of combination anti-retroviral therapies (cART), it is now estimated that more than half of HIV-infected individuals will develop some form of HAND [14], highlighting the need for effective therapies to address this continual burden. Indeed, cognitive impairment can occur despite minimal viral load [14], [15], as low-level viral replication occurs even with the most effective anti-retroviral regimens [16]–[19], giving rise to early pro-inflammatory viral proteins, such as the transactivator of transcription (Tat), that persist even in the presence of cART. This has led to the widely accepted notion that the development of HAND may be ascribable to the migration of inflammatory monocytes into the CNS following their activation in the periphery in response to multiple host cell-derived products and pro-inflammatory viral proteins [20]–[22]. Thus, exploring the mechanisms by which the BBB becomes permeabilized during infection will reveal new potential targets, thereby aiding in the development of therapeutic interventions.

In the current report, we demonstrate that the HIV protein Tat delivered systemically is able to induce platelet activation *in vivo*, leading to an increase in plasma sCD40L concentrations. Tat treatment also resulted in CD40L-dependent augmentation of BBB permeability, while complementary intravital multiphoton analysis revealed increased adherence of leukocytes to the brain microvasculature in wild-type mice, but not CD40L deficient mice, in the presence of Tat. Collectively, these data shed light on the mechanisms of CNS infiltration during HIV infection.

## Materials and Methods

### Ethics Statement

All experiments involving the use of laboratory animals were carried out in accordance with the Animal Welfare Act and the National Institute of Health (NIH) guidelines, and the animal protocol was approved by the University Committee on Animal Resources of the University of Rochester Medical Center. The facilities and programs of the Vivarium and Division of Laboratory Animal Medicine of the School of Medicine and Dentistry are fully accredited by the Association for the Assessment and Accreditation of Laboratory Animal Care International (AAALAC) and are in compliance with state law, federal statute, and NIH policy. All animals used in these studies, strains C57BL/6 and B6.129s2-CD40lg^tm1Imx^/J, were purchased from The Jackson Laboratory, Bar Harbor, ME.

### Reagents and Antibodies

HIV Tat_1–72_ was obtained from Dr. Avindra Nath (National Institute of Neurological Disorders and Stroke, Baltimore, MD) and Philip Ray (University of Kentucky, Lexington, KY). Production of this protein has been described previously [23]–[25] and the purified protein was found to be >98% pure, with less than 1 pg/mg endotoxin content of Tat protein [23]–[25]. We have also tested the effects of heat inactivated Tat (H.I. Tat) that was prepared in the same manner, but subsequently incubated at 85°C for 30 minutes, as previously described [26], [27], as this allows a control for nonspecific contaminants that may arise during production and processing of the protein. We found no significant differences between treatment with H.I. Tat and saline controls, and therefore, in some cases saline controls alone are displayed. It should also be noted that when working with Tat all instruments were siliconized with Sigmacote (Sigma-Aldrich, St. Louis, MO) to avoid loss of the reagent.

Fluorescein sodium salt was purchased from Sigma-Aldrich (St. Louis, MO); recombinant mouse CD40L was purchased from R&D Systems (Minneapolis, MN). Antibodies against mouse granulocyte antigen 1 (Gr1; also known as Ly-6C/G) conjugated to Alexa Fluor 488, and Texas Red Dextran were obtained from Invitrogen (Carlsbad, CA), while phycoerythrin-conjugated anti-mouse CCR-2 was purchased from R&D Systems (Minneapolis, MN). Antibodies for platelet depletion, as well as non-immune rat immunoglobulin control antibodies, were purchased from Emfret Analytics (Eibelstadt, Germany).

### ELISA

Ten to twelve-week old wild-type C57BL/6 (WT) mice (n = 5 for each group) were injected retro-orbitally with HIV Tat (100 ng/g body weight). One-hour post-injection, whole blood was collected via cardiac exsanguination and sequentially centrifuged to obtain platelet poor plasma (PPP). Soluble CD40L or platelet factor 4 (PF4) concentrations were measured in PPP samples using either a mouse sCD40L or PF4 ELISA kit (R&D Systems, Minneapolis, MN) according to the manufacturer’s protocol. Samples were compared using an unpaired t-test with statistical significance indicated in the figure as **p<0.01 and ***p<0.001.

### Tail Bleed Assays

Ten to twelve-week old WT mice (n = 5 for each group) were treated with saline, Tat (100 ng/g body weight), or H.I. Tat (100 ng/g body weight) that was injected retro-orbitally. One-hour following treatment, mice were anesthetized and placed on a raised platform with tails protruding over the edge. Tails were positioned 5 mm above filter paper and a 2 mm cut was made in the tip of the tail. Time was recorded from the moment the cut was made until bleeding stopped completely.

### Complete Blood Counts

For each experiment in which complete blood counts were performed, mice were bled from the retro-orbital sinus and 20 µL of whole blood was collected into glass capillary tubes coated with EDTA. Counts were then performed using a Heska CBC-Diff Veterinary Analyzer (Fort Collins, CO). Platelet counts from the same animals taken on subsequent days were compared using paired t-tests, with significance indicated in the figure as *p<0.05, **p<0.01, and ***p<0.001.

### Sodium Fluorescein Assay

Ten to twelve-week old male C57BL/6 WT or female CD40L deficient (homozygous; C57BL/6 background; CD40L KO) mice (n = 6 for each group) were injected retro-orbitally with HIV Tat (1 µg/g body weight), while control mice were injected with saline. Twenty-three hours later, mice were injected intraperitoneally (i.p.) with the fluorescent tracer sodium fluorescein (10 mg/mL in 200 µL PBS) for 1 h. Mice were then anesthetized with an i.p. injection of a ketamine (100 mg/kg) and xylazine (10 mg/kg) cocktail, following which, whole blood was collected via cardiac exsanguination. Platelet poor plasma was obtained following sequential centrifugation, as previously described [28]. Following blood collection, animals were perfused with 30 mL cold PBS through the left ventricle. Tissues were harvested and subsequently homogenized in cold PBS (1∶10 weight per volume), subjected to precipitation in 15% trichloroacetic acid, and pH was adjusted using NaOH. Fluorescence in prepared tissues or plasma was read using a SpectraMax M3 Multimode Microplate Reader (Molecular Devices, Sunnyvale, CA) with excitation at 485 nm and emission at 530 nm. Permeability was determined as the ratio of brain fluorescence/plasma fluorescence for each animal and samples were analyzed as fold change compared to saline treated WT animals.

### Reverse Transcription Polymerase Chain Reaction (rtPCR)

In an effort to verify the absence of CD40L in the CD40L deficient animals, as previously reported [29]–[31], spleen homogenates were prepared using tissue collected from both wild-type and CD40L deficient animals. Subsequently, total RNA was isolated using an RNeasy Mini Kit, according to the manufacturer’s protocol (Qiagen, Valencia, CA). First strand complementary DNA synthesis was then performed using the Invitrogen SuperScript III First-Strand Synthesis System (Invitrogen, Carlsbad, CA), and rtPCR was then completed using the Platinum *Pfx* DNA polymerase (Invitrogen, Carlsbad, CA) and the following primers for mouse CD40L: 5′- TGAAATGCAAAGAGGTGATGAGGA-3′ (forward) and 5′- GAGCCCAGGTCAACCATAACAGAT-3′ (reverse). The following primers specific for the housekeeping gene GAPDH were also used as a positive control: (forward) 5′-TGATGACATCAAGAAGGTGGTGAA-3′ and (reverse) 5′-TCCTTGGAGGCCATGTAGGCCAT-3′.

### Recombinant sCD40L Assays

Ten to twelve-week old WT mice (n = 4 for each group) were injected i.p. with recombinant mouse sCD40L (0.2 µg/g body weight) that was resuspended in PBS with 0.1% BSA, or with saline. Twelve hours post-injection, sodium fluorescein assays were performed as detailed above. Recombinant sCD40L can be injected both intraperitoneally [32] or directly into the bloodstream [33]; we have tested both methods of delivery in our model and obtained similar results regardless of the method used.

### Platelet Depletion

Ten to twelve-week old WT mice (n = 6 for each group) were injected retro-orbitally with antibodies for platelet depletion or non-immune rat immunoglobulin control antibodies (0.5 µg/g body weight of either type; Emfret Analytics, Eibelstadt, Germany). The antibodies for platelet depletion are a mixture of purified rat monoclonal antibodies that target the GP1bα receptor found on platelets and result in rapid Fc-independent platelet depletion [34], [35]. Twenty-four hours post-depletion, complete blood counts were performed to verify loss of platelets and ensure all other counts remained within the normal range. Following the counts, saline or Tat (1 µg/g body weight) was injected into the retro-orbital sinus of the opposite eye. Complete blood counts were once again performed after an additional 24 h, and sodium fluorescein assays were performed as described above. Platelet poor plasma was also used to perform sCD40L ELISAs (R&D Systems, Minneapolis, MN), according to the manufacturer’s protocol. Platelet counts from the same animals taken on subsequent days were compared using paired t-tests, with significance indicated in the figure as *p<0.05, **p<0.01, and ***p<0.001.

### Intravital Multiphoton Imaging

Ten to twelve-week old male WT or female CD40L KO mice (n = 3 for each group) were injected retro-orbitally with HIV Tat (1 µg/g body weight), while control mice were injected with saline. Twenty-four hours post-treatment, mice were anesthetized with isoflurane (1 – 1.5%) and ventilated through a facemask. Subsequently, animals were injected through the femoral vein with fluorescently conjugated antibody directed against granulocyte antigen 1 (Gr1; aka Ly-6C/G) for visualization of leukocytes (monocytes, neutrophils), and Texas Red Dextran for illumination of vessels. Body temperature was maintained using a water-flow heating pad (Gaymar, Orchard Park, NY) and a temperature controller (WPI, Inc., Sarasota, FL), and cortical windows were then prepared as previously described [36]–[38]. Time-lapse videos of cortical venules were captured using XY images taken every 4 seconds for 400 frames at depths up to approximately 200 microns with a Spectra Physics MaiTai HP Deep See Olympus Fluoview1000-AOM multiphoton imaging system (Olympus America, Inc., Center Valley, PA). For imaging, we used a 25X NA 1.05 water-immersion objective (Olympus XLPlan N) and recorded on Fluoview1000 software (Olympus America, Inc., Center Valley, PA); images were taken at 12-bit depth resolution of 512×512 pixels with a pixel dwell time of 2 microseconds. For each imaging experiment, integrity of the cortical window site was verified under brightfield illumination and arterioles and venules were identified based on blood flow direction, branch patterns, and color under white light. Videos were then analyzed, 2 fields per animal, and quantitation was performed in a random, unbiased fashion by counting visible adhered and labeled cells in each field.

### Flow Cytometry

Animals (n = 3) were treated as in the multiphoton imaging experiments, and 24 h post-treatment whole blood was obtained via cardiac exsanguination. Whole blood was stained using Alexa Fluor 488-conjugated antibody directed against granulocyte antigen 1 (Gr1; aka Ly-6C/G) and with phycoerythrin-conjugated anti mouse CCR-2. Samples were read on an Accuri C6 Flow Cytometer (Accuri Cytometers, Ann Arbor, MI) and 15,000 leukocytes per sample were collected. Analysis was performed using FlowJo Flow Cytometry Imaging Software (FlowJo, Ashland, OR) and cells were gated based on Gr1 expression. Subsequently, Gr1 high cells were then used to determine the percentage of CCR-2 expression within the Gr1 positive population. Fluorescence minus one controls were used for analysis and samples were compared as percent positive for expression of either Gr1 or CCR-2.

### Statistical Analysis

For each experiment, unless otherwise noted, statistical significance was determined using one-way ANOVA followed by Bonferroni’s test for multiple comparisons. Data from each replicated experiment is represented as mean ± SEM for each group with statistical significance indicated in each figure as *p<0.05, **p<0.01, and ***p<0.001.

## Results

### HIV Tat Activates Platelets in vivo, Thereby Inducing Excess sCD40L Release

**Figure 1:**
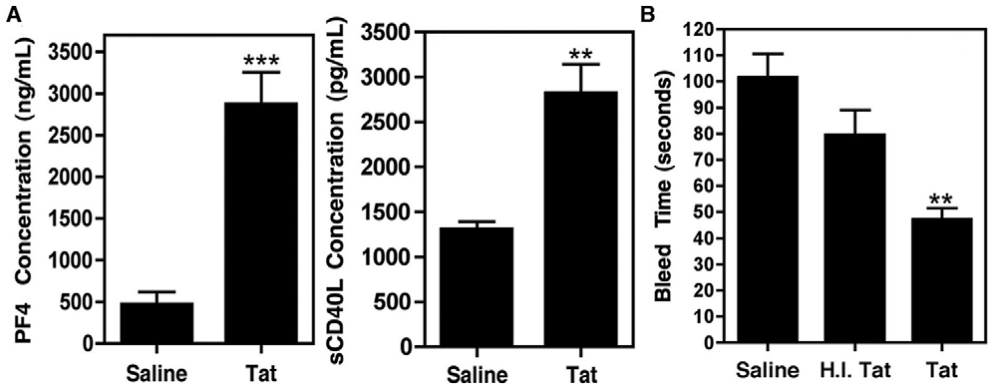
HIV Tat activates platelets *in vivo.* (**A**) Plasma concentrations of platelet factor 4 (PF4) and soluble CD40L (sCD40L) in wild-type C57BL/6 (WT) mice were measured via ELISA. Upon injection with HIV Tat (100 ng/g body weight; n = 5), there is a significant increase in the level of each of these platelet-derived mediators in the plasma after 1 h, indicating that Tat stimulates platelets *in vivo*. Samples were compared using an unpaired t-test with statistical significance as **p<0.01 and ***p<0.001. (**B**) Tat significantly decreased the time to clot in WT mice that had been nicked in the tail, indicating platelet activation. Tat that had been heat inactivated (H.I. Tat) prior to treatment did not significantly reduce bleeding time as compared to saline treatment. Samples were compared via one-way ANOVA followed by Bonferroni’s test for multiple comparisons, which indicated statistical significance as **p<0.01 for the Tat treated animals compared to both saline and H.I. Tat.

As previously mentioned, we have shown that increased levels of sCD40L are found in the plasma and CSF of HAND patients as compared to HIV-infected, non-cognitively impaired individuals [8]. Therefore, in an effort to create a simplified model of HIV-induced inflammation *in vivo* that would allow us to focus on platelet activation, we injected HIV Tat into wild-type C57BL/6 (WT) mice and subsequently analyzed markers of platelet activation. A single, low dose (100 ng/g body weight; n = 5), retro-orbital injection of Tat induced platelet activation, resulting in a significant release of the pro-inflammatory mediators platelet factor 4 (PF4) and sCD40L after just one hour (Figure 1A). Furthermore, Tat treatment led to a reduction in clot time (Figure 1B), as determined using tail bleed assays, indicative of platelet activation. This was not seen in mice treated with the same dose of Tat that had been heat inactivated (H.I. Tat) prior to treatment (Figure 1B), which was used as a control to ensure that the observed effects were not the result of byproducts, including endotoxins, created during the production of Tat. In addition, intraperitoneal (i.p.) injection of lipopolysaccharide (LPS; 1 µg/g of body weight) in WT mice did not stimulate the release of sCD40L (data not shown), further indicating that the observed effect of Tat on platelets is not the result of endotoxin contamination from production and processing of the protein. This dose and method of administration of LPS was chosen so as to avoid endotoxic shock, as previously validated [39]. Collectively, these results suggest that a single injection of Tat is sufficient to stimulate platelet activation *in vivo.*

### HIV Tat Increases Blood Brain Barrier Permeability

**Figure 2:**
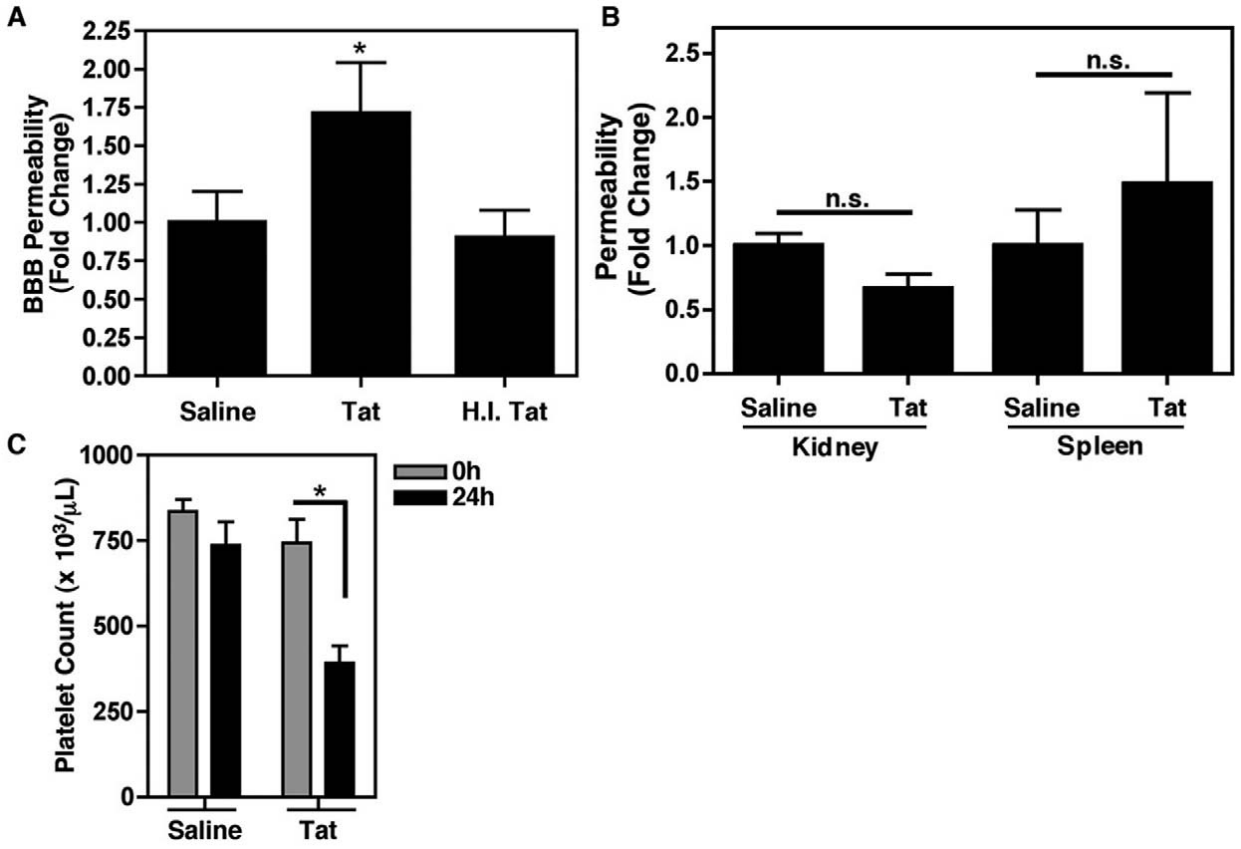
Tat increases blood brain barrier (BBB) permeability. (**A**) Wild-type C57BL/6 (WT) mice were treated with Tat (1 µg/g body weight; n = 6) for 24 h. The fluorescent tracer sodium fluorescein (NaF) was then used to assess BBB permeability. HIV Tat, but not heat inactivated Tat (H.I. Tat), significantly increased BBB permeability as compared to saline treated animals. The values are presented as fold increase in the ratio of brain versus plasma concentrations of NaF. (**B**) The Tat-induced increase in vascular permeability was limited to the BBB in our model since there was no increase in fluorescence in other tissues, such as the spleen or kidney, in the same WT animals. Groups were compared in both (A) and (B) using one-way ANOVA followed by Bonferroni’s test for multiple comparisons with statistical significance indicated as *p<0.05 or n.s. as not significant. (**C**) Complete blood counts indicated that there was a Tat-induced drop in platelet count by 24 h, indicative of activation followed by consumption of these cells. Groups were compared individually using a paired t-test with significance indicated as *p<0.05.

In an effort to examine whether Tat-induced inflammation would lead to altered BBB permeability, we next employed the sodium fluorescein (NaF) assay. Sodium fluorescein is commonly used as a marker of BBB permeability since it can only cross the barrier paracellularly once it has been compromised; therefore, by determining the ratio of brain fluorescence to plasma fluorescence for each animal, we can determine the degree of BBB permeability following a given treatment. In our model, Tat, but not heat inactivated Tat, significantly increased BBB permeability 24 h post-treatment, as compared to saline alone (Figure 2A), to levels consistent with those previously reported [40]. This effect was limited to the brain, as both the kidneys and spleen demonstrated no significant increase in permeability, as measured in our model, following Tat treatment (Figure 2B). Complete blood counts were also performed on saline and Tat treated animals and it was found that Tat treatment led to a significant reduction in platelet count 24 h post-treatment (Figure 2C), suggestive of platelet activation followed by clearance in these animals. This data also lends to the idea that the mechanism behind HIV-induced thrombocytopenia is indeed loss of platelets subsequent to aberrant activation [41], [42].

### CD40L is Required for Tat-induced BBB Permeability

**Figure 3:**
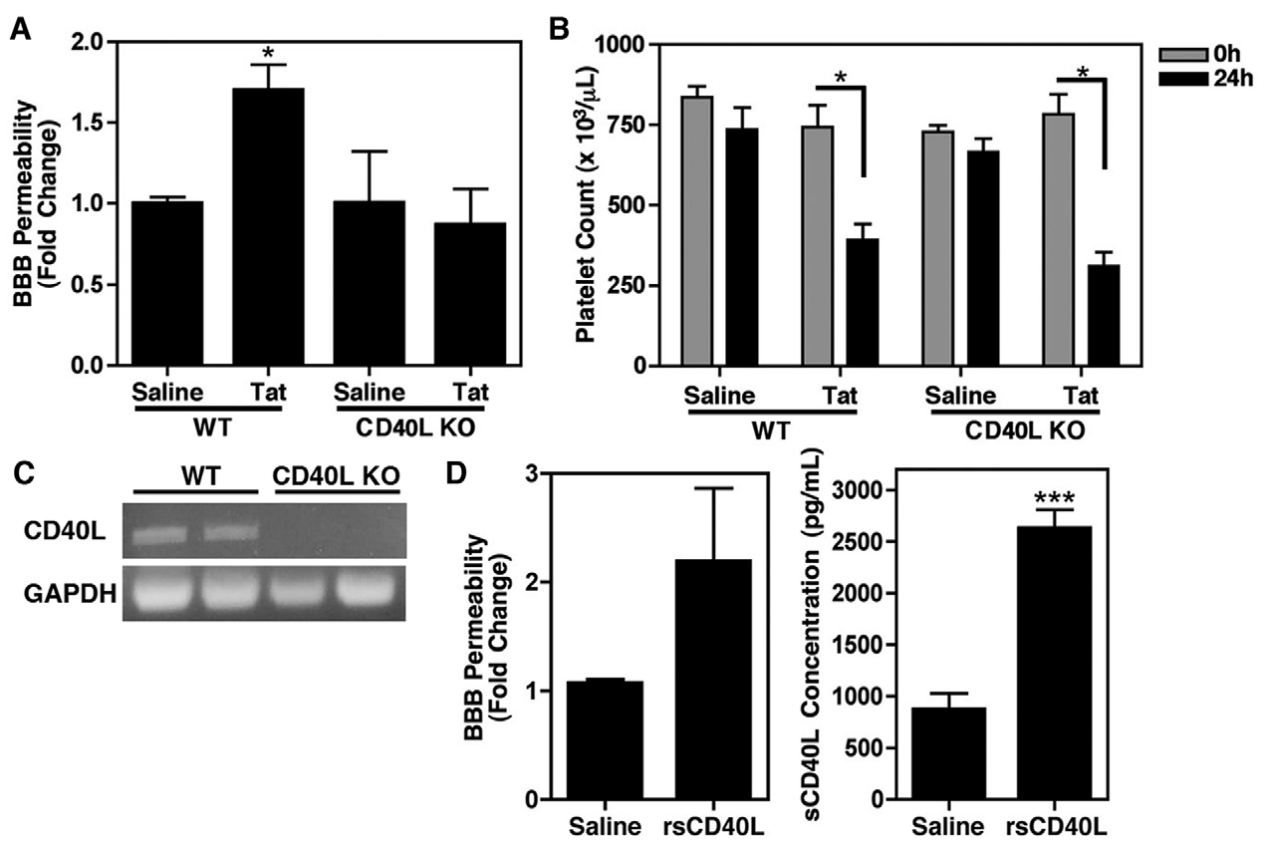
CD40L is required for Tat-induced BBB permeability. (**A**) Wild-type C57BL/6 (WT) or CD40L deficient (CD40L KO) mice were injected retro-orbitally with HIV Tat (1 µg/g body weight; n = 6 per group), while control mice were injected with saline. Sodium fluorescein (NaF) analysis revealed that the Tat-induced increase in BBB permeability is dependent on CD40L. The values shown here represent fold increase in the ratio of brain versus plasma concentrations of NaF. (**B**) Complete blood counts reveal that both WT and CD40L KO animals demonstrate a Tat-induced drop in platelet count by 24 h post-treatment. For panels (A) and (B) statistical significance was determined via one-way ANOVA followed by Bonferroni’s test for multiple comparisons and indicated in the figure as *p<0.05. (**C**) Verification that CD40L expression is absent in CD40L KO animals. Representative results obtained from reverse transcription-PCR of spleen homogenates using primers specific for CD40L and GAPDH. (**D**) Recombinant mouse sCD40L (rsCD40L; 0.2 µg/g body weight) was injected intraperitoneally into WT animals (n = 4). Twelve hours post-treatment NaF assays were performed and it was revealed that rsCD40L induced similar BBB permeability as treatment with Tat alone. An unpaired t-test indicated that p = 0.0531. sCD40L ELISA confirmed that there was a significant increase in circulating sCD40L following treatment with rsCD40L (right panel), and an unpaired t-test determined statistical significance as ***p<0.001.

CD40L has been implicated in several inflammatory disorders involving altered BBB permeability, including ischemia/reperfusion injury [43] and severe malaria [31]. Thus, in an effort to determine whether the accumulation of sCD40L that is seen in HAND patients could be contributing to BBB permeability during HIV infection, thereby leading to increased infiltration of the CNS by activated leukocytes, we next repeated the NaF assays in mice deficient in CD40L (CD40L KO). Experiments were performed as described above, with a single dose of Tat injected retro-orbitally 24 h prior to performing the NaF assay. In WT animals, Tat induced a significant increase in BBB permeability, however this effect was completely abolished in CD40L KO animals (Figure 3A). Complete blood counts revealed that CD40L KO animals also demonstrated the same Tat-induced drop in platelet count as WT animals following the 24 h treatment (Figure 3B), indicative of platelet activation. This would suggest that other platelet derived pro-inflammatory releasates would still be present in the circulation of these animals, thus highlighting the importance of CD40L in Tat-induced BBB permeability. In an effort to verify that CD40L was indeed absent in the KO animals, reverse transcription-PCR was also performed using spleen homogenates prepared from both WT and CD40L KO animals, as the spleen is known to store monocytes, lymphocytes, and platelets [44]–[46], and it was determined that CD40L is indeed absent in these animals (Figure 3C).

To further demonstrate that the Tat-induced increase in sCD40L is contributing to augmented BBB permeability, we performed alternative NaF assays in which recombinant mouse sCD40L (rsCD40L) was injected intraperitoneally in place of Tat. This method of delivery resulted in significant increases in plasma sCD40L concentrations that were detectable within 30 minutes (data not shown) and lasted up to 12 h post-injection (Figure 3D). Furthermore, injection of sCD40L alone produced a similar increase in BBB permeability as that seen using Tat treatment (Figure 3D), further implicating sCD40L in the pathogenesis of HAND.

### Platelets are Required for Tat-induced BBB Permeability

**Figure 4:**
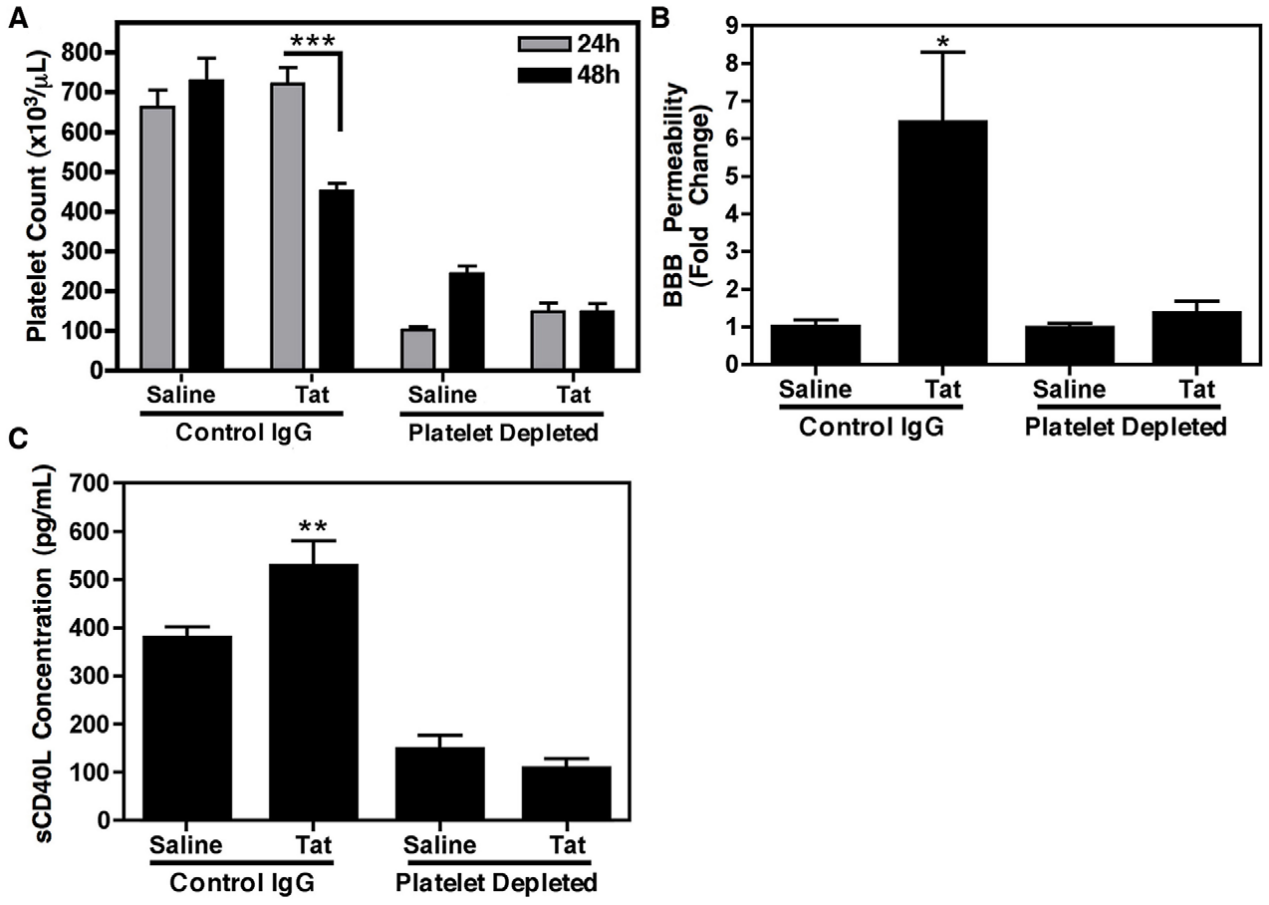
Platelet-derived sCD40L is contributing to Tat-induced BBB permeability. (**A**) Complete blood counts verified efficient platelet depletion in wild-type C57BL/6 (WT) animals (n = 6) 24 h following treatment with antibodies for either platelet depletion or control, non-immune rat immunoglobulin (Control IgG; 0.5 µg/g body weight of either antibody). Tat treatment (1 µg/g body weight) also led to a significant loss of platelets in control antibody treated, but not platelet depleted, animals (at 48 h post-depletion, 24 h following Tat treatment). Legend indicates time of complete blood counts post antibody injection. (**B**) Sodium fluorescein analysis of animals treated in (A) revealed that platelets are required for the Tat-induced increase in BBB permeability. (**C**) Whole blood was collected via cardiac exsanguination from animals treated as in (A) and ELISA specific for sCD40L was performed on platelet poor plasma samples. As expected, ELISA analysis revealed a Tat-induced increase in sCD40L in animals that had been treated with control antibodies; however in animals that had been depleted of platelets, concentrations were lower then saline treated control animals, indicating that platelets are the major source of circulating sCD40L. In panels (A−C) samples were compared via one-way ANOVA followed by Bonferonni’s test for multiple comparisons with statistical significance indicated as *p<0.05, **p<0.01, and ***p<0.001.

Since platelets are widely believed to be the main source of sCD40L, estimated to produce approximately 95% of all circulating sCD40L [3], and we have now demonstrated that Tat induces an increase in plasma concentrations of sCD40L in our model, we next determined whether the presence of platelets was required for Tat-induced BBB permeability. To do so, we employed platelet-depleting antibodies, which are a mixture of purified rat monoclonal antibodies that target the GP1bα receptor found on platelets, and result in rapid Fc-independent platelet depletion [34], [35]. Non-immune rat immunoglobulin was also used as a control antibody. Platelet depletion was performed in WT mice 24 h prior to injection of Tat and complete blood counts were performed to verify depletion. Approximately 75–80% depletion was achieved, measured immediately before Tat injections, and this was maintained 24 h following Tat treatment (Figure 4A; 48 h timepoint). As expected, Tat treated animals that received control antibodies demonstrated a significant decrease in platelet count following treatment. Consistently, Tat induced a significant increase in BBB permeability in control antibody treated animals (Figure 4B), as measured using the NaF assay. However, this effect was completely abolished when platelets were depleted prior to Tat treatment, suggesting that platelet activation, and subsequent sCD40L release, are indeed playing a role in BBB permeability in our model (Figure 4B).

Since it is well established that several cell types in addition to platelets, such as T cells and B cells, express CD40L and possess the ability to shed this molecule from their surface upon stimulation [47], we also sought to verify that platelets were the major source of the Tat-induced sCD40L detected in the plasma of these animals. Following platelet depletion, we saw a significant reduction in the amount of sCD40L present in the plasma of Tat treated animals as compared to control immunoglobulin, Tat treated animals (Figure 4C). Additionally, control animals displayed the same pattern of Tat-induced sCD40L stimulation as presented above, while even saline treatment in the animals that had been depleted of platelets demonstrated concentrations well below the baseline levels seen in the control immunoglobulin animals. Collectively these data demonstrate that platelets are the major source of sCD40L found in circulation.

### HIV Tat-induced Inflammation Promotes Leukocyte Attachment to Brain Microvascular Endothelial Cells in WT, but not CD40L Deficient, Animals

**Figure 5:**
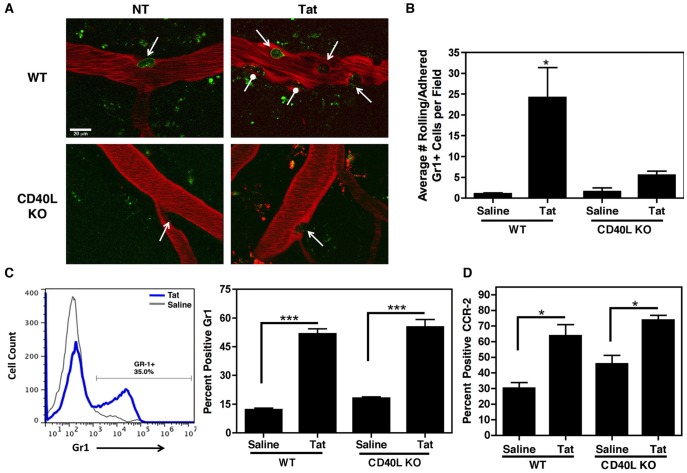
Tat increases the number of rolling and adhered leukocytes to the brain microvasculature. (**A**) Representative images of cortical two-photon time-lapse videos in wild-type C57BL/6 (WT) or CD40L deficient (CD40L KO) mice (n = 3). Tat (1 µg/g body weight) was injected retro-orbitally 24 h prior to creation of cortical window and subsequent imaging. The cerebral blood vessels were fluorescently labeled with Texas Red Dextran that was injected into the femoral vein prior to acquisition. Leukocyte rolling and adhesion was visualized using Alexa Fluor 488-conjugated antibody against the granulocyte antigen 1 (Gr1) that was injected in the same manner. Tat treatment in WT, but not CD40L KO, mice induced an increase in the number of rolling (arrows) and adhered (circles) Gr1 positive cells. (**B**) Quantitation of Gr1 positive cells rolling on or adhered to vessels in the two-photon time-lapse videos. (**C**) WT or CD40L KO animals were treated as in (A) and whole blood was collected via cardiac exsanguination. Subsequent flow cytometric analysis of monocytes using the same fluorescently labeled anti-Gr1 antibody (left panel shows representative Gr1 positive gating) detected an equal number of Gr1 positive, inflammatory monocytes in both WT and CD40L KO animals. (**D**) Samples described in (C) were also analyzed using fluorescently labeled anti-CCR-2 to monitor chemokine receptor expression in response to Tat in both WT and CD40L KO mice. (B–D) Values in each panel were compared using one-way ANOVA followed by Bonferroni’s test for multiple comparisons with statistical significance defined as *p<0.05 and ***p<0.001.

We recently demonstrated that treatment of BMVECs with CD40L resulted in increased adhesion and migration of monocytes in an *in vitro* model of the BBB [9]. To determine whether Tat treatment, and subsequent inflammation *in vivo,* leads to an increased number of leukocytes that roll along and adhere to the brain microvasculature, we next performed intravital multiphoton image analysis in WT and CD40L KO mice that had been treated with Tat. Twenty-four hours post treatment, cortical windows were created and time-lapse videos were captured at depths up to approximately 200 microns. Leukocytes (monocytes, neutrophils) were labeled using fluorescently conjugated antibody directed against granulocyte antigen 1 (Gr1; aka Ly-6C/G), as murine monocytes expressing high levels of Gr1 have been shown to be representative of the “inflammatory” monocyte population that corresponds to the CD16 high monocyte subset within humans [48]–[50], and the Gr1 antigen is also representative of a widely used panel of markers for activated monocytes in mice [51]–[53]. These mice were also exposed to Texas Red Dextran for the illumination of vessels. Quantitation of rolling and adhered Gr1 positive cells (representative images derived from time-lapse videos are shown in Figure 5A) revealed that Tat treatment significantly increases the number of these inflammatory cells on venules within the cortex of WT mice (Figure 5B) as compared to treatment with saline. In contrast, Tat treated CD40L KO animals demonstrated a significant decrease in the number of Gr1 positive migrating cells as compared to WT Tat treated animals, suggesting that Tat’s effect is primarily CD40L-dependent.

Complementary flow cytometric analysis confirmed that an equal number of Gr1 positive leukocytes were present in both WT and CD40L KO animals following exposure to Tat (Figure 5C), indicating that the leukocytes within the CD40L KO animals are still able to be stimulated in the presence of Tat, however, they no longer roll along or adhere to the microvasculature. Furthermore, Tat-induced chemokine receptor expression, as measured by CCR-2 expression, in Gr1 positive leukocytes remained unchanged between WT and KO animals (Figure 5D), indicating that there is no defect in chemokine response in the KO animals. Taken together, these data highlight the importance of CD40L signaling within BMVECs in Tat-induced leukocyte infiltration of the CNS.

## Discussion

In light of the recent observation that more than half of HIV-infected individuals will develop some form of neurocognitive impairment regardless of anti-retroviral therapy [14], it seems apparent now more than ever that novel therapeutic interventions are lacking and in dire need. Disruption of the BBB contributes to the pathogenesis of many neurological disorders [13], [54] and is widely regarded as a large contributing factor in the pathogenesis of HAND [11], [12], [55]. In the current study, we investigated whether the accumulation of sCD40L seen in HAND patients [8] could be playing a role in BBB permeability, thus aiming to identify new targets to aid in the development of therapeutics for these disorders. We now report that treatment of C57BL/6 mice with HIV Tat alone is sufficient to cause platelet activation, coupled with a significant increase in plasma sCD40L concentration and an increase in BBB permeability.

Consistent with the data reported herein, Tat has previously been reported to induce platelet activation [56]. Interestingly, we previously demonstrated that treatment of purified platelets with Tat alone did not stimulate the release of sCD40L from these cells, measured via ELISA, indicating that Tat does not directly activate platelets [8]. However, we now demonstrate that treatment *in vivo* with Tat leads to the release of sCD40L from platelets, indicating that Tat exerts an indirect effect on platelet activation. Consistently, it has been reported that Tat upregulates molecules known to activate platelets, such as platelet activating factor [57], [58], derived largely from monocytes, which would not be present in the *in vitro* setting. On the contrary, Wang et al. (2011) recently observed that Tat induces direct platelet activation, ultimately leading to the upregulation of surface expressed CD40L on platelets, as measured via flow cytometry [56]. This discrepancy with our previous report [8] may be clarified when considering that Wang et al. measured surface expressed CD40L, whereas we measured the soluble form of this molecule. Therefore, direct treatment of Tat on purified platelets may in fact lead to an upregulation of CD40L on the surface of platelets, as reported [56], however, there may be other factors required to stimulate the release of CD40L from the surface of platelets in order to generate the soluble form, which may be absent in the *in vitro* setting, however present *in vivo*. Indeed, the mechanism of release of CD40L from the surface of platelets has yet to be fully elucidated [59], [60], though it is known to be a complex process involving numerous intracellular pathways [59] that may not be fully stimulated following treatment of platelets *in vitro* with Tat alone.

HAND is widely considered to be an inflammatory disorder, while CD40L has been previously implicated in a variety of other inflammatory disorders [3], [61], including those associated with neuroinflammation [31], [43], [62]–[64]. Interestingly, several of these studies demonstrate that attenuation of CD40/CD40L signaling ameliorates disease-associated neuroinflammation [31], [43], [65], further highlighting the involvement of the CD40/CD40L axis in inflammatory disorders of the CNS. To date, there have been several reports implicating a role for this receptor/ligand pair in the pathogenesis of HAND [8], [9], [66], however its exact role, while presumably multi-factorial, remains largely unknown. Interestingly, it seems that the ability of the BBB to respond to sCD40L is high; as Ramirez et al. (2010) have demonstrated that the receptor for CD40L, CD40, is highly expressed on brain endothelial cells of HAND patients [9]. In the same report it was demonstrated that exposure of primary human brain microvascular endothelial cells to sCD40L leads to increased expression of adhesion molecules, ICAM-1 and VCAM-1, in a cJun-N-terminal kinase (JNK) dependent manner [9]. These results are consistent with those previously reported in which ligation of CD40 by platelet-derived CD40L was able to induce an inflammatory phenotype of endothelial cells, thus inducing upregulation of ICAM-1 and VCAM-1, as well as the release of chemokines interleukin-8 (IL-8), a potent neutrophil chemoattractant, and monocyte chemoattractant protein-1 (MCP-1) [67]. Interestingly, Chakrabarti et al. (2007) observed that the release of MCP-1 from sCD40L-stimulated endothelial cells is a redox-dependent mechanism resulting from reactive oxygen species (ROS)-induced NF-κB activation [68], while CD40 ligation on endothelial cells has previously been shown to stimulate ROS generation [69], [70]. Although the exact mechanism has yet to be elucidated, nitric oxide (NO) has also been previously implicated in BBB permeability [71], [72], however CD40L-induced ROS generation has been shown to antagonize endothelial NO production [69], [70]. It is noteworthy that Chen et al. (2008) observed that sCD40L treatment of human coronary endothelial cells led to alterations in mitochondrial membrane potential and a decrease in ATP levels, which was reversed following treatment with an antioxidant [69], and accordingly, it has been demonstrated that maintenance of tight junction permeability, especially in the context of the BBB, is controlled by tyrosine phosphorylation and ATP levels [72]–[74]. Thus, we postulate that CD40L-induced alterations in endothelial cell signaling may be contributing to increased BBB permeability by altering tight junction maintenance and increasing the attraction and adhesion of activated leukocytes to the BBB.

Here we demonstrate using CD40L deficient mice that CD40L signaling is necessary for HIV Tat-induced BBB permeability, collectively suggesting that the accumulation of sCD40L may be contributing to inflammatory disorders of the CNS by allowing pro-inflammatory leukocytes access to the brain, a tightly regulated organ which they may not have otherwise gained access to. On the contrary, we do not see increased permeability within other tissues in our model, suggesting that this effect is limited to the brain. However, it is known that HIV infection is associated with increased systemic inflammation [75]–[77], and thus, it is probable that there is increased inflammation within the vasculature of other organs upon Tat treatment in our model, though the baseline fluorescence in these tissues is likely higher to begin with, due to more frequent paracellular transport of the endothelial cell layers of these organs than that of the BBB [78]. Given the highly specialized regulation of the tight junctions between BMVECs [78], [79], it is likely that there is less paracellular transport of sodium fluorescein under normal conditions at the BBB than in other tissues, and thus upon stimulation, we are able to detect the increased fluorescent signal within the brain, but not other tissues, in our model.

Injection of recombinant Tat protein alone is an attractive and relevant feature of our model, since cognitive impairment can occur despite minimal viral load [14], [15], [21], while many viral factors would not be present; commonly attributed to low-level viral replication that occurs even with the most effective anti-retroviral regimens [16]–[19], [21] giving rise to early pro-inflammatory viral proteins, such as Tat, that persist even in the presence of cART [80]. Thus, Tat would continue to be secreted from infected cells at various locations throughout the body, and consequently give rise to an activation cascade that would contribute to a sustained pro-inflammatory state in the periphery. Indeed, it has been demonstrated previously that even transient exposure of macrophages to Tat can create a sustained inflammatory response [81]. Along the same lines, the concentration of Tat used in our model is likely an underestimate of the local concentrations that neighboring cells would be exposed to as Tat is secreted during HIV infection. Although the concentration of Tat has been measured in the sera of HIV-infected patients [82], the authors note that this is likely an underestimate of local concentrations that bystander cells would be exposed to upon secretion from neighboring infected cells [82]. Tat can be taken up by nearly every cell type in the body [83], thus creating a very short half-life *in vivo*, making it extremely difficult to determine actual local concentrations of this protein that bystander cells would be exposed to, or estimate a physiologically relevant concentration of Tat to use in experimental models [27], [84]–[86]. Therefore, it is common among many groups, including our own, to use higher doses of recombinantly produced protein, consistent with those employed in the current manuscript, to sufficiently mimic the effects of Tat that would be present locally during HIV infection [27], [40], [84]–[87].

Furthermore, as discussed, Tat treatment induces a pro-inflammatory state that triggers an activation cascade in which more platelets would activate over time upon exposure to more and more inflammatory mediator release; however, CD40 is expressed on a wide variety of cell types [61] and it is likely that a majority of the sCD40L would be consumed shortly after its release from platelets. Therefore, it would not be expected that a single, low dose of Tat delivered peripherally would lead to sustained sCD40L release at concentrations high enough to stimulate widespread endothelial cell activation. Accordingly, a single, low dose injection of Tat (100 ng/g body weight) induced stimulation of platelets that occurred very quickly, as we could measure increases in plasma sCD40L concentrations as soon as 1 h post-treatment (Figure 1), however, this increase was not sustained over time and thus, this dose was not sufficient to induce BBB permeability (data not shown). Therefore, we employed a high-dose, single injection strategy (1 µg/g body weight) that resulted in sustained increases in sCD40L concentrations that lasted at least 24 h post-treatment (Figure 5). This acute exposure to Tat also mimics the waxing and waning nature of HAND, as the secretion of Tat would not be constant during infection, but varies during the course of the disease. Furthermore, unpublished data from our lab demonstrate that a similar concentration of Tat injected into the tail vein of mice was able to induce plasma levels of Tat comparable to those reported in the sera of HIV-infected patients [82]. This higher concentration is also consistent with that used by Chen et al. (2009) in which they observe a similar Tat-induced increase in BBB permeability as that seen in our model, and comparable to that employed by Lu et al. (2011) in which they inject Tat directly into the hippocampus of mice and observe durable microgliosis that lasts up to 28 days post-injection. Thus, we feel our model is well suited to measure Tat-induced changes in inflammation and subsequent BBB permeability, as it relates to HIV-mediated inflammatory changes that are observed in HIV-infected patients. It is also noteworthy that our model may predict early consequences of HIV infection that may cumulatively contribute to long term chronic effects, such as those demonstrated by Lu et al.

Evidence suggests that sCD40L is not the only pro-inflammatory molecule contributing to increased stimulation and permeability at the BBB in the context of HIV infection, as TNFα [88] and HIV gp120 [89]–[91] have both previously been reported to alter BBB integrity. Consistently, we do not see a complete reversal of Tat’s effect on adherence of Gr1 positive leukocytes in the absence of CD40L (Figure 5B). However, our multiphoton and complementary flow cytometric analysis demonstrate that CD40L KO animals have decreased adherence of leukocytes to BMVECs in the presence of Tat, while their Gr1 and CCR-2 expression remain unchanged as compared to WT animals (Figure 5). As previously mentioned, in mice, Gr1 high monocytes are comparable to the CD16 high inflammatory monocyte subset within humans [48], which is expanded during HIV infection [92], while CCR-2 is the chemokine receptor for monocyte chemoattractant protein-1 (also known as CCL-2) previously demonstrated to be important for migration of leukocytes across an *in vitro* model of the BBB in the context of HIV [93]. Collectively these data indicate that leukocytes in the KO animals retain their ability to become stimulated, increasing Gr1 expression in response to Tat, and are able to respond to increased chemokine signals, increasing CCR-2 expression. This would suggest that they simply no longer bind to BMVECs in the CD40L KO animals or, in other words, that the BMVECs are no longer receptive to the activated leukocytes. Considering the upregulation of adhesion molecules on BMVECs in response to excess sCD40L reported by Ramirez et al. (2010), it is likely that the absence of CD40L signaling in these animals alters adhesion molecule upregulation in response to HIV-associated effector molecules, thereby reducing the attachment and subsequent extravasation of leukocytes through BMVECs. Thus, while other pro-inflammatory molecules in addition to sCD40L are undoubtedly playing a role in activation and increased permeability of the BBB, inhibiting CD40/CD40L signaling may prove to be a beneficial point of intervention in the pathogenesis of HAND.

Targeting of CD40L signaling is a complex issue, given the importance it plays in stimulation of antigen presenting cells, and thus inhibition could greatly alter immune competence and humoral immune responses [94], [95]. For this reason, current strategies for inhibition of CD40L, such as anti-CD40L antibodies, may have the ability to induce immunosuppression and hence would not be suited for use in HAND. However, anti-platelet agents that have the ability to reduce overall plasma concentrations of sCD40L, such as valproic acid (VPA) [28], would be advantageous, as they would allow for control of sCD40L release from platelets without interfering with surface expressed, co-stimulatory CD40L on other cell types. Indeed, VPA has shown promise in initial clinical trials for controlling pathologic aspects of HAND in HIV-infected patients [96]. This is especially relevant in light of the current data that platelets do indeed seem to be the primary source of a majority of the circulating sCD40L, and are necessary for Tat-induced BBB permeability (Figure 4). This data is also consistent with the notion that activated platelets and successive consumption, ultimately leading to decreased platelet count or thrombocytopenia, can be predictive of brain injury and disease progression [6], [7], [97].

Collectively these results solidify the notion that the CD40/CD40L axis plays a crucial role in determining BBB integrity and vulnerability to activated leukocytes. Furthermore, platelet activation during infection may also be playing a critical role in the pathogenesis of HAND. Therefore, targeting sCD40L may prove to be a worthy avenue of pursuit in developing novel therapeutic interventions not only for the management of HAND, but also for various other inflammatory disorders in which CD40L has been implicated.
